# The influence of friends and siblings on the physical activity and screen viewing behaviours of children aged 5–6 years: a qualitative analysis of parent interviews

**DOI:** 10.1136/bmjopen-2014-006593

**Published:** 2015-05-14

**Authors:** M J Edwards, R Jago, S J Sebire, J M Kesten, L Pool, J L Thompson

**Affiliations:** 1 Centre for Exercise, Nutrition & Health Sciences, School for Policy Studies, University of Bristol, Bristol, UK; 2 School of Sport, Exercise and Rehabilitation Sciences, University of Birmingham, Birmingham, UK

**Keywords:** PUBLIC HEALTH, QUALITATIVE RESEARCH, Physical activity, Sedentary activity

## Abstract

**Objectives:**

The present study uses qualitative data to explore parental perceptions of how their young child's screen viewing and physical activity behaviours are influenced by their child's friends and siblings.

**Design:**

Telephone interviews were conducted with parents of year 1 children (age 5–6 years). Interviews considered parental views on a variety of issues related to their child's screen viewing and physical activity behaviours, including the influence that their child's friends and siblings have over such behaviours. Interviews were transcribed verbatim and analysed using deductive content analysis. Data were organised using a categorisation matrix developed by the research team. Coding and theme generation was iterative and refined throughout. Data were entered into and coded within N-Vivo.

**Setting:**

Parents were recruited through 57 primary schools located in Bristol and the surrounding area that took part in the B-ProAct1v study.

**Participants:**

Fifty-three parents of children aged 5–6 years.

**Results:**

Parents believe that their child's screen viewing and physical activity behaviours are influenced by their child's siblings and friends. Friends are considered to have a greater influence over the structured physical activities a child asks to participate in, whereas the influence of siblings is more strongly perceived over informal and spontaneous physical activities. In terms of screen viewing, parents suggest that their child's friends can heavily influence the content their child wishes to consume, however, siblings have a more direct and tangible influence over what a child watches.

**Conclusions:**

Friends and siblings influence young children's physical activity and screen viewing behaviours. Child-focused physical activity and screen viewing interventions should consider the important influence that siblings and friends have over these behaviours.

**Strengths and limitations of this study**

- The study provides new information on an important but relatively underexplored public health topic; the influence that friends and siblings have physical activity (PA) and screen viewing (SV) behaviours of children aged over 5–6 years.
- Recruitment of a purposive sample of participants from a large sample enabled us to collect data from families with a range of different levels of PA and SV, from different deprivation groups.
- The qualitative data provide a deeper understanding (than that provided in quantitative studies) of the influence that friends and siblings have PA and SV behaviours of children aged over 5–6 years.
- The study is limited by the exclusive use of parental reports on sibling and friend influences. As children spend a large period of time in school and parents cannot observe behaviour or influence in this setting, children and/or teachers’ views would be valuable to obtain (particularly for PA, as much SV is done in the home).

## Introduction

Regular physical activity (PA) is associated with lower risk of overweight and obesity, reduced adiposity, beneficial effects on lipid and triglyceride levels, improved cardiovascular fitness, reduced anxiety and depressive symptoms, and improvements in children's academic performance.^1^ National surveys in the UK and elsewhere indicate that many children fail to meet government recommendation of at least 60 min of moderate-to-vigorous-intensity PA (MVPA) each day.^2^ ^3^ As such, there is a need to increase child PA levels.

Screen viewing (SV; ie, television, computer, console, tablet computer and mobile phone use) has become a ubiquitous form of sedentary behaviour among children and adolescents.^4^ High levels of SV have been associated with a number of negative health effects including a higher likelihood of being overweight,^5–10^ reduced mental well-being,^11^ acute coronary syndrome^12^ and other cardiovascular risk factors.^13^ Social issues that have been associated with high SV include a heightened risk of irregular nap-time and sleep-time schedules for young children,^14^ delinquent behaviour and social problems,^15^ ^16^ reduced time engaged in active play, and less time spent with parents and siblings in other activities.^17^

The American Academy of Pediatrics recommends that children's total SV time be limited to 1–2 h/day.^18^ However, data from the 2012 Health Survey for England found that average sedentary time—watching television, reading, eating, studying, drawing, using computers^19^—for children (aged 2–15) was 3.2 h for girls and 3.3 for boys on weekdays (outside of school) and 4 h for girls and 4.2 h for boys on weekend days. Close to half of this time was spent viewing television (1.5–2.2 h for girls and 1.5–1.8 h for boys, dependent on age).^19^ Owing to high levels of child SV and the negative health and social consequences associated with such behaviours, there is a strong need to reduce child SV time. Reducing SV time in childhood is given added importance as sedentary behaviours have been found to track from childhood to adulthood.^20^

Recent evidence suggests that friendship groups play an important role in shaping children's PA.^21–27^ Systematic reviews of social network influence on childhood PA found strong similarities in the PA behaviour of individuals within friendship groups^22^ and that a child's PA was positively associated with that of their friends.^23^ ^28^ Some research suggests that the closer a friendship bond is, the greater the influence on PA is likely to be.^27^ ^29^ Of equal importance, however, may be belonging to a diverse range of friendship groups.^21^ A report by de la Haye *et al*^30^ suggests that the influence of friends over SV behaviours may have a gendered dimension, with girls playing a more influential role on other girls than is the case for boys.

The association between friendship groups and sedentary activities is more ambiguous than that for PA.^23^ Ali *et al*^31^ found no peer effect on the television viewing behaviour of US adolescents. However, drawing from the same data set (Add Health), Fletcher^32^ found that the number of hours a child views television is determined, in part, by their peers in school (not necessarily ‘friends’). An hour increase in the school-level average television viewing time was associated with a ∼30 min increase in individual television viewing time (95% CI 0.06 to 0.82).

Although a burgeoning body of research on PA and SV considers the impact of friends, there is a relative lack of attention given to the effect that child siblings have on the PA behaviours of one another. As children spend a large amount of time with their siblings, it is important to consider sibling influence. There is a limited amount of research in this area. Sallis *et al*^33^ found the influence of siblings to be consistently related to adolescent PA. Hohepa *et al*^24^ identified an association between a child's after-school PA activity participation and their sibling's (with a stronger association for younger students). There is also mixed evidence for sibling influence on child SV. Bagley *et al*^34^ found that boys without siblings spent more time watching television (153.2 min/day) compared with those with siblings (129 min/day). Girls with siblings spent less time viewing television (127.1 min/day) than those without siblings (134.8 min/day).^35^ Having a sibling has also been associated with a twofold increase in the likelihood of adolescents viewing ≥2 h of television per day (OR=2.2, 95% CI 1.0 to 5.2).^35^

The existing literature is limited, as it mainly focuses on the impact of friends on PA and SV among adolescents,^23^ ^26^ and there is little research into the impact of friendship networks on the PA and SV behaviours of *young* children.^36^ ^37^ In addition, the majority of previous research is quantitative and, while valuable, qualitative research would facilitate a deeper understanding of the influence friends and siblings may have over children's PA and SV behaviours. As parents of young children are central to their child's life and facilitate or restrict much of their child's interaction with siblings and friends, they are likely to be a valuable source of information. Thus, the aims of the present study were to (A) explore parental opinions on the influence their 5–6 -year-old child's friends have over their child's SV and PA behaviours, and (B) explore parental opinions on the influence their child's sibling have over their child's SV and PA behaviours.

## Methods

Participants were recruited from the B-ProAct1v study. Full details of the sampling and recruitment methods have been published elsewhere.^38^ ^39^ Briefly, B-ProAct1v is a large cross-sectional study that aims to identify factors that are associated with PA and SV among year 1 children (aged 5–6, in their second year of school). Children (n=1267) and at least one of their parent(s) wore an accelerometer (Actigraph GT3x+) for 7 days and completed a questionnaire that assessed parent (8 items) and child (6 items) SV. Parents reported the amount of time that they and their child spent using TV, computer/laptop and consoles, on weekdays and weekends (less than 1, 1–2, 2–3, 3–4, 4–5, 5 h or more). In addition, parents reported their smartphone, tablet or PC usage. Participating parents also gave consent to be invited to participate in a semistructured interview at a later date. This study draws on the qualitative data produced through these interviews.

Parents of children who provided ≥3 valid days of accelerometer wear time (480 min, to allow estimation of PA level), and an address and postcode (to allow for calculation of socioeconomic position (SEP)), were included in the sampling frame for interviewing. A random stratified sampling method was used to ensure varying degrees of SEP, based on Index of Multiple Deprivation (IMD) score^40^ and a range of active to inactive children (based on time spent in MVPA). This procedure was conducted by using thirds of MVPA and IMD scores to calculate ‘low’, ‘medium’ and ‘high’ MVPA/SEP categories, creating a three by three matrix (9 groups). People were randomly selected (by computer algorithm) from the different matrix categories and an attempt was made to recruit an equal number of participants to be interviewed from each category. This was done in order to achieve diversity in the PA and deprivation levels of participants. Of the 1267 parents who completed a questionnaire, 990 (78.1%) consented to be contacted to take part in an interview. In total, 274 participants were randomly selected and invited to participate in an interview. Of these, 53 parents (19.3%) agreed to take part.

The 53 parents (49 mothers, 4 fathers) who took part in in-depth interviews received £10 gift vouchers. Written informed consent was obtained for all interview participants.

A semistructured interview guide was developed by the research team at the start of the study, with questions revised iteratively throughout the data collection process (based on emergent issues gained from the interviews). At the start of each interview, parents were reminded of the purpose of the study and asked to comment only in relation to the child taking part in the study (and not other children they may have). Interviews considered parental views on a variety of issues, including the following: (A) general impression of child PA and SV behaviours; (B) parental concerns over the amount of child PA and SV; (C) whether their child's SV and PA behaviour was influenced by others including friends and siblings; and (D) where, when and with whom their child engaged in SV and PA. Interviews were recorded using a digital recorder (Olympus DS-3400) and transcribed verbatim. There were 633 pages of transcribed text (font size 10, single spaced, Verdana). Transcripts were compared with audio files to check for accuracy (researchers listened to all audio recordings and compared these to the transcribed text. Any inaccuracies (place names, eg) in the transcribed text were edited appropriately). The average length of interviews was 25 min (range 12–50 min).

## Data analysis

Interviews were analysed using deductive content analysis.^41^ During the preparation, phase transcripts were read several times by two or more researchers to immerse themselves in the data. The units of analysis were themes identified from the literature. Data were then organised using a categorisation matrix (see Methods section for more details) developed by the research team. For the present study, any data relating to friend and sibling influence over PA and SV were abstracted. Coding and theme generation was iterative and refined throughout. There were frequent peer debriefings to enhance the accuracy of reporting the data and to critically question the findings. All queries and conflicts over the meaning of the content and reporting of data were discussed through a process of triangulation until a consensus was reached between study staff (RJ, SJS, JLT, MJE, LP and JMK). Study staff met at regular intervals throughout the analysis phase to discuss emergent issues and themes. Issues that were of particular interest, or those in need of greater consideration, were discussed between the group until a consensus was reached as to how data should be interpreted and reported. There were no major disagreements over the interpretation of data.

Data were entered into and coded within N-Vivo (V.10, QSR, Southport, UK). Hierarchies of categories were created and summarised, and brief summaries and representative quotes for each category were abstracted for reporting purposes. The quotes were selected as they are illustrative of several responses given by parents. Results are presented alongside details of child sex, objectively measured child MVPA per day (low, medium, high), level of SEP (low, medium, high) and average hours of self-reported weekday+weekend day SV estimate (SV/d) for the study child. This information is presented in order to provide greater contextual depth. Categorisation of MVPA and SEP into ‘low’, ‘medium’ and ‘high’ was achieved by dividing the respective data into tertiles.

## Results

The average age of parents was 37.5 years (SD=5.92). Eighty-nine per cent of participants had two or more children and 86% of the sample was white British. Twenty-three per cent were unemployed or full-time parents with 77% in full-time or part-time work. The data reflect parental beliefs about their child's behaviour. It is not an account of what has actually happened. The themes that are explored below emerged from the interview data. The results are divided into two sections. The first section explores the influence of friends and siblings on young children's PA behaviours. The second section focuses on the influence that friends and siblings have on children's SV behaviour.

### Friend influence on participation in PA

Twenty-three parents stated that their child's PA was influenced by friends. Parents perceived that their children's awareness of PA opportunities and engagement in these activities were influenced by friends (largely from school). Parents reported that the greatest influence exerted by their child's friends on PA was shaping the desire to attend structured PA sessions. Little direct influence from friends over informal or impromptu PA was mentioned.

Yeah, gymnastics, that was her idea from school then tennis was [an] idea from school. So yeah I would say that other children influence her on that. (Female, Medium SEP/Medium MVPA, 4 SV/d)

I think when he decided that he wanted to do rugby and gym, I think that was influenced because of friends already doing it. (Male, Low SEP/Medium MVPA, 3.5 SV/d)

Parents also stated that the influence of friends can exceed the actual appeal of activities themselves, resulting in children attending activities they dislike.

If his friends are doing something then he comes in and he is first to tell me that, oh his friends are joining Judo, he wants to. So I set him up with Judo, he goes for one session and that's it, yes, so there's another five to follow and he just won't go. He will cry and what have you. So, yeah, but his friends do influence him yes, if his friends are doing something then he will come and tell me that he wants to do it or try it. (Male, High SEP/Low MVPA, 2 SV/d)

He does swimming lessons, which quite a few of his friends do […] he started doing it and absolutely hated it. He's getting on quite well with his swimming lessons now. He kind of goes along and he does enjoy them but he often does complain before going that he doesn't want to go to swimming. (Male, Low SEP/Medium MVPA, 3 SV/d)

Ten parents suggested that their child's PA choices are not influenced by friends. These parents often cited their child's strong personality as a reason for this. All comments made relating to children having strong personalities were attributed to daughters.

She doesn't mind what other people are doing because she knows what she likes. What she likes, yes, and she could be influenced but only if she already knew that she wanted to do it anyway […] she is not that bothered what other people are doing. (Female, High SEP/Medium MVPA, 1 SV/d)

She's quite single-minded. I mean, her ballet she does away from school—there are no children from her school where she does ballet. (Female, Medium SEP/High MVPA, 2 SV/d)

Four parents claimed that a mismatch in their child's and their child's school friends’ PA preference was a reason for little friend influence. In other cases, a lack of neighbourhood friends restricted engagement in PA.

In his school, in his year group there's 60 children and there's no other boy that plays football […] They all play rugby, all his best friends. So I mean he wants to play football, that's what he wants to do […] He's gone to a club, he's made separate friends, he didn't know anyone at the club and the same with his karate. No one else [friends from school] does that. (Male, Low SEP/High MVPA, 4.5 SV/d)

We live on [a] quiet little estate on the corner, and there's mostly boys in the street that play cricket and do things in the cul-de-sac. So she doesn't really have people to play with like that. So she does her own thing. (Female, Medium SEP/Low MVPA, 2.5 SV/d)

### Sibling influence on PA

Thirteen parents stated that their child's PA is influenced by their sibling. Sibling influence on PA was largely referred to in the form of informal and impromptu activity—including cycling, playing in the garden and riding a scooter—as opposed to structured and/or fee paying activities. An exception appeared to be when siblings were particularly skilled at an activity. Only one parent felt that siblings did not influence PA behaviour.

They [siblings] go out in the garden and she will go and play with them, I suppose so. She doesn't tend to want to play on her own. (Female, Medium SEP/High MVPA, 1 SV/d)

[My child] will follow [his sibling] outside if [his sibling] decides that he wants to go and play on his scooter or something. If he's out, then even if [my other child] is kind of saying ‘no I'm reading a book’, he'll kind of eventually drift off and follow him and then, yeah, and they'll both run around chasing one another. (Male, Low SEP/Medium MVPA, 3 SV/d)

Parents thought that older siblings exerted an ‘aspirational influence’ whereby younger brothers and sisters seek to mirror their achievements. When siblings are particularly good at an activity, this sometimes prompted interest in the organised PAs their sibling wished to do. There was little difference in how parents referred to sibling influences of boys and girls, however, older siblings tended to influence the younger more often (although younger siblings do at times influence older siblings’ PA).

Her brother is very sporty, very, and I think because he is very good at what he does, I think she wants to be like him. (Female, Medium SEP/Medium MVPA, 2.5 SV/d)

In fact, I had to always pull her back because she looks up to her brother who is a very active boy and he has been going to all these things, and she always wanted to be just like him. (Female, High SEP/Medium MVPA, 1 SV/d)

### Friend influence on SV

Twelve parents suggested that the television programmes and films their child views are influenced by friends. However, friends are seen to have little *direct* influence over viewing habits. The role friends play can be described as more akin to ‘opinion forming’ (influencing what children ask to view) than ‘behaviour shaping’ (what children actually consume), although the former may result in the latter. Some content that children are influenced by friends to view is against the wishes of parents and/or disliked by the child when consumed. The most often-cited influence that friends exert—although still subject to parental approval—is over remote online gaming.

The only thing that [the respondent's child has] come home with is Club Penguin [online multiplayer game], the one you play on the PC, that's the only thing that he's sort of been influenced by other people. And Skylanders [video game] […] and Moshi Monsters [online game], which is just like, you know, the old sort of game that you play on the computer. (Male, Medium SEP/High MVPA, 2.5 SV/d)

In one sense, a lot of his friends have this Skylanders game…And [my child] kept saying ‘oh mummy can we get Skylanders, can we get Skylanders?’ And I said ‘no, because we can't afford it’. As simple as that. And then we were at his cousins and they've got Skylanders, so they put it on, and he was, he was terrified of it, and has never asked since. (Male, Low SEP/High MVPA, 3 SV/d)

Eight parents suggested that their child's SV behaviour is not affected by friends. Overwhelmingly, children who are not influenced by friends were seen as having strong personality characteristics, meaning they ‘made their own choices’, without pressure from friends. In contrast to PA, there is little difference in parental comments made in relation to male or female SV.

He's quite strong willed really, he will do what he wants. I can't think of a situation where that [child's SV being influenced by friends] would have happened. (Male, Medium SEP/High MVPA, 3 SV/d)

She's quite single-minded. If she likes a programme and nobody else does like it, I don’t think it would bother her much. She's not one to necessarily run with the crowd. (Female, Medium SEP/High MVPA, 2 SV/d)

He knows what he likes and, and that's it really. (Male, Low SEP/Medium MVPA, 2 SV/d)

### Sibling influence on SV

Twelve parents reported that their child's SV behaviour is influenced by siblings. Parents reported that older children influenced younger sibling's SV behaviour, in terms of total viewing time and content. Sibling co-viewing of television programmes and films was commonly reported and largely fortuitous, often with one child having the television on and the other joining in. Only two parents stated that their child's SV behaviour was not influenced by siblings.

Her oldest brother probably influences her a bit more on the screen stuff, because he's on the computer all the time when he's staying at our house…So she's probably copying him a bit more with that, rather than her friends. (Female, High SEP/High MVPA, 3.5 SV/d)

It's more of a problem with his brother really, I have to limit DS time for his brother and [the younger child] […] he does sometimes do DS himself, but he does quite like curling up with his [older] brother and watching his brother doing the DS. (Male, Medium SEP/Low MVPA, 3 SV/d)

The influence older siblings have over SV can be seen as problematic and coercive, with parents sometimes having to intervene.

They've [older siblings] probably got a greater influence. More so because they’re, they’re trying to convince him that he wants to do something that they actually want to do…So we do tend to get that and I do have to sort of step in with, ‘Er, excuse me, that isn't actually appropriate for [your brother]’. (Male, High SEP/High MVPA, 3.5 SV/d)

If we have a power struggle here, it comes from him [child's older brother] and occasionally it comes from the lips of the girls, but I know that he is standing there and just, you know, he sends his little sister and [she] says to me “mummy can I watch tele?” And then she tells me what it is she wanted to watch, and by ‘tele’ she means a DVD, and by the choice I know exactly that it was [her brother's choice]. (Female, High SEP/Medium MVPA, 1 SV/d)

## Discussion

The data presented in this paper suggest that the majority of parents believe their child's PA and SV to be influenced by their siblings and friends. For some parents, there was a feeling that their child's PA and SV behaviour were not influenced by others. Such parents made statements describing their child as slightly different—‘single-minded’, ‘strong willed’—to other children. On reflection, however, the findings suggest that, combined, friends and siblings can have an important effect on many young children's PA and SV choices and behaviours. There was little difference, for PA or SV, in relation to the comments made regarding male or female children.

In line with previous qualitative research in children aged 10–11 years and adolescents,^21^ ^42^ parents perceived their child's friends to affect the initiation of PA. Past quantitative research has found evidence for similar PA behaviours within friendship networks, and research suggests that friends’ PA is a strong predictor of an individual's PA.^22^ In the present study, structured PA choices (most often fee paying classes) were perceived to be influenced by ‘school’ friends (opposed to ‘neighbourhood’ or ‘other’ friends^21^). In several cases the child who adopted the PA preferences of their friend(s) was thought to actually dislike the activity when attempted. This suggests that the motivation for some PA choices may stem from a desire to spend time with or engage in similar behaviours to others rather than a desire to participate in the PA through inherent interest or enjoyment.^42^ Siblings were seen to influence home-based PA, be it in the garden or neighbourhood, but played less of an influence over structured PA (the exception being when older siblings inspired the younger). Similar to previous research,^24^ this suggests that there may be a temporal dimension to the influences over PA, with siblings being more influential outside of school hours. As children appear to be strongly influenced by friends in their PA choices, provision of opportunities for companionship or playmates outside of school hours, as suggested by Hesketh *et al*,^43^ may be an effective way to increase PA among friendship groups. Additionally, greater parental awareness of free, informal and easily accessible opportunities^44^ may help facilitate increased PA with siblings and with friends.

Parents believed that friends and siblings play a complementary and important role in young children's SV behaviour. Our findings suggest that friends play a more pronounced role in influencing the desires of children, whereas siblings—due largely to the domestic nature of much SV—directly influence viewing behaviour. Such observations have not been reported elsewhere, but are of importance when designing interventions aimed at decreasing SV time in children. Depending on the setting and aim of an intervention, material/content may be more effective if designed with the most relevant stakeholders in mind. For example, an intervention aimed at reducing after-school hours SV in the home may have a greater likelihood of success if sibling influence is targeted. The data also suggest that friends play an important role in a burgeoning strand of SV, remote online gaming, with young children frequently enquiring about and/or accessing such technology. As the average UK citizen now owns more than 11 media devices,^45^ it is essential that researchers widen their scope of focus to explore the impact of a widening array of new media devices^46^ on children's SV practices. Similarities in friends’ video, computing and internet usage were found by de la Haye *et al*,^30^ but they did not explore online gaming.

Several parents felt that their child's SV and/or PA choices were not influenced by friends or siblings. In the majority of cases, parents stressed that their child had particularly strong personality traits that rendered them impermeable to friend influence. This is an interesting finding, as much research suggests that friends and siblings do influence child PA and SV behaviour.^21–27^ ^32^ ^33^ For PA, only girls were mentioned as not being influenced, however, there was no gender difference mentioned in terms of SV.

## Implications of findings

To our knowledge, this is the first study to suggest that the influence friends have over young children's PA is largely through opinion forming, as opposed to directly influencing the actual process of children being physically active. While siblings appear to play a less significant role in terms of opinion forming, their influence over the content of, and total time spent, SV, was seen as greater by parents. Together, parents consider friends and siblings to play an important role in the PA choices and behaviours of young children. Interventions aimed at reducing SV behaviour and/or increasing PA levels among young children should incorporate both friends and siblings. Research should further explore the unique influence of friends and siblings.

## Strengths and limitations of this study

The study provides new information on a relatively underexplored topic. A strength of the study is the recruitment of a purposive sample of participants from a large sample, which has enabled us to collect data from families with a range of different levels of PA and SV from different deprivation groups. The study is limited by the exclusive use of parental reports on sibling and friend influences. A relatively low response rate (19.3%) was achieved, however, this was deemed to provide a sufficient and feasible (within staff and funding restrictions) number of participants (n=53) for the purpose of the qualitative phase of the study. Additionally, there was a low proportion of male views (7.6%). This may have resulted in an unbalanced set of findings, favouring the views of mothers. While male views are prominently featured in the findings, future studies may wish to adopt a recruitment strategy aimed at achieving a more gender-balanced sample.

As children spend a large period of time in school and parents cannot observe behaviour or influence in this setting, children and/or teachers’ views would be valuable to obtain (particularly for PA, as much SV is done in the home). A further limitation of the study is that no formal analysis of the different matrix groups was conducted in relation to SV or PA. Such analysis was beyond the scope of the present paper.

## Conclusions

Parents perceive their 5–6 -year -old child's friends and siblings to play an important role over the SV and PA choices and behaviours their child makes. Attempts to reduce child SV time and/or increase their PA levels may be more successful if developed with friends’ and siblings’ influential role in mind.
